# A biomechanics dataset of healthy human walking at various speeds, step lengths and step widths

**DOI:** 10.1038/s41597-022-01817-1

**Published:** 2022-11-16

**Authors:** Tim J. van der Zee, Emily M. Mundinger, Arthur D. Kuo

**Affiliations:** 1 University of Calgary, Biomedical Engineering Graduate Program, Calgary, Canada; 2 University of Calgary, Faculty of Kinesiology, Calgary, Canada

**Keywords:** Physiology, Biophysics

## Abstract

The biomechanics of human walking are well documented for standard conditions such as for self-selected step length and preferred speed. However, humans can and do walk with a variety of other step lengths and speeds during daily living. The variation of biomechanics across gait conditions may be important for describing and determining the mechanics of locomotion. To address this, we present an open biomechanics dataset of steady walking at a broad range of conditions, including 33 experimentally-controlled combinations of speed (0.7–2.0 m·s^−1^), step length (0.5–1.1 m), and step width (0–0.4 m). The dataset contains ground reaction forces and motions from healthy young adults (N = 10), collected using split-belt instrumented treadmill and motion capture systems respectively. Most trials also include pre-computed inverse dynamics, including 3D joint positions, angles, torques and powers, as well as intersegmental forces. Apart from raw data, we also provide five strides of good quality data without artifacts for each trial, and sample software for visualization and analysis.

## Abstract (summary)

**Table:** 

Measurement(s)	ground reaction force • body position
Technology Type(s)	force platform • motion capture
Factor Type(s)	walking speed • step frequency • step length • step width

## Background & Summary

Walking is the primary means of moving about in the environment, yet the biomechanics have been described only for relatively standard conditions. In daily living, humans may adjust gait variables according to conditions, for example step length to cross a set of paving stones, or step width to traverse a narrow path. Furthermore, humans use different step lengths and step widths when navigating uneven terrain^1^, which may have implications for energy expenditure^2–5^. Experimental data of healthy walking across a broad range of conditions could help to describe how these adjustments occur, and may inform musculoskeletal- and other biomechanical models of locomotion.

We here present a biomechanics dataset of healthy human walking, covering a broad range of walking conditions. The dataset contains biomechanical variables for 33 combinations of speed (0.7–2.0 m·s^−1^), step length (0.5–1.1 m), and step width (0–0.4 m), varied in five types of conditions. It contains ground reaction forces and motions from healthy young adults (N = 10, age = 23.5 ± 2.5), collected using split-belt instrumented treadmill and motion capture systems respectively (for 60 s per trial). Most trials also contain 3D joint positions, forces, angles, torques and powers, obtained from inverse dynamics analysis on the lower limbs using standard software.

Portions of this data have been used in previously published studies. These include studies of redirection of the body center of mass^6^ and of work performed by soft tissues during both preferred^7^ and varied step length walking conditions^8^.

## Methods

### Participants

Ten healthy young adult participants (age 23.5 ± 2.5 years, body mass 73.5 ± 15 kg, height 1.76 ± 0.11 m, mean ± standard deviation) were enrolled in the experiments. Table 1 provides anthropometric information on the included participants. All participants provided their informed consent to participate in the experiment, which complied with all relevant ethical regulations. Both informed consent and study protocol were approved by the Institutional Review Board of the University of Michigan, where the experiment was performed.

**Table 1:** Anthropometric information of included participants.

	Age	Body mass	Height	Leg length	Foot width	Pref. step length s*	Pref. step freq. f*	# Raw files	#5strides files
p1	25 yrs.	81.8 kg	1.73 m	0.89 m	0.13 m	0.71 m	1.77 Hz	33	28
p2	21 yrs.	57.3 kg	1.64 m	0.94 m	0.09 m	0.60 m	2.07 Hz	33	28
p3	23 yrs.	97.5 kg	1.90 m	1.04 m	0.11 m	0.71 m	1.77 Hz	33	27
p4	21 yrs.	57.0 kg	1.73 m	0.88 m	0.10 m	0.68 m	1.83 Hz	33	27
p5	21 yrs.	56.7 kg	1.63 m	0.86 m	0.09 m	0.65 m	1.93 Hz	33	28
p6	24 yrs.	72.6 kg	1.83 m	0.94 m	0.10 m	0.74 m	1.70 Hz	33	26
p7	28 yrs.	86.2 kg	1.89 m	0.99 m	0.10 m	0.74 m	1.70 Hz	32	27
p8	27 yrs.	88.6 kg	1.85 m	0.99 m	0.10 m	0.74 m	1.70 Hz	33	28
p9	22 yrs.	77.0 kg	1.78 m	0.94 m	0.10 m	0.68 m	1.83 Hz	33	27
p10	23 yrs.	60.3 kg	1.60 m	0.86 m	0.10 m	0.65 m	1.93 Hz	33	0

Leg length was measured from floor to greater trochanter. Preferred step length s* and step frequency f* are for walking at 1.25 m·s^−1^. #Raw files and #5strides files indicate the number of available trials for the raw C3D data, and the 5 strides data respectively.

### Experimental protocol

Participants walked on an instrumented treadmill at 33 different combinations of average walking speed $\bar{v}$, step length *s*, step frequency *f*, and step width *w*. Ground reaction forces and motions were recorded for 60 s of steady-state gait for each combination. There were five sets of constraints (see Table 2), where some gait parameters were experimentally varied (“Variable”) and some were fixed (“Fixed”) between conditions: *Preferred walking* at various average walking speeds $\bar{v}$, *Variable step length walking* at several step lengths *s* but fixed step frequency *f*, *Variable speed walking* at several step frequencies *f* but fixed step length *s*, *Fixed speed walking* with inversely varying combinations of step length *s* and step frequency *f*, or *Variable step width walking* at several step widths *w* but fixed speed $\bar{v}$ and fixed step frequency *f*. Step length *s* and step frequency *f* were varied relative to the individual preferred values s*and f*, determined from unconstrained walking at a nominal speed (v* = 1.25 m·s^−1^). The nominal walking speed was chosen based on previous research^9^, suggesting that 1.25 m·s^−1^ approximately corresponds to the preferred walking speed for adults. Walking speed $\bar{v}$ and step frequency *f* were manipulated by setting the treadmill belt speed and asking participants to walk on the beat of an audio cue, respectively. Step length *s* was manipulated through both walking speed and step frequency from their ratio $s=\bar{v}/f$. Step width *w* was self-selected in all conditions except *Variable step width walking*, where it was experimentally manipulated by asking participants to step on laser lines projected onto the treadmill surface at the specified widths. Participants generally followed experimental directions reasonably well but imperfectly, and so actual step lengths, frequencies, and widths should be obtained from the data.

**Table 2:** Trial lookup.

Trial #	Speed	Step length	Step frequency	Step width	Walking condition
1	0.70 m·s^−1^	1.00 s*	0.56 f*	Self-selected	Variable speed
2	0.70 m·s^−1^	Self-selected	Self-selected	Self-selected	Preferred walking
3	0.70 m·s^−1^	0.56 s*	1.00 f*	Self-selected	Variable step length
4	0.90 m·s^−1^	1.00 s*	0.72 f*	Self-selected	Variable speed
5	0.90 m·s^−1^	Self-selected	Self-selected	Self-selected	Preferred walking
6	0.90 m·s^−1^	0.72 s*	1.00 f*	Self-selected	Variable step length
7	1.10 m·s^−1^	1.00 s*	0.88 f*	Self-selected	Variable speed
8	1.10 m·s^−1^	Self-selected	Self-selected	Self-selected	Preferred walking
9	1.10 m·s^−1^	0.88 s*	1.00 f*	Self-selected	Variable step length
10	1.60 m·s^−1^	1.28 s*	1.00 f*	Self-selected	Variable step length
11	1.60 m·s^−1^	Self-selected	Self-selected	Self-selected	Preferred walking
12	1.60 m·s^−1^	1.00 s*	1.28 f*	Self-selected	Variable speed
13	1.80 m·s^−1^	1.44 s*	1.00 f*	Self-selected	Variable step length
14	1.80 m·s^−1^	Self-selected	Self-selected	Self-selected	Preferred walking
15	1.80 m·s^−1^	1.00 s*	1.44 f*	Self-selected	Variable speed
16	2.00 m·s^−1^	Self-selected	Self-selected	Self-selected	Preferred walking
17	1.25 m·s^−1^	1.43 s*	0.70 f*	Self-selected	Fixed speed
18	1.25 m·s^−1^	1.25 s*	0.80 f*	Self-selected	Fixed speed
19	1.25 m·s^−1^	1.11 s*	0.90 f*	Self-selected	Fixed speed
20	1.25 m·s^−1^	1.00 s*	1.00 f*	Self-selected	Multiple conditions
21	1.25 m·s^−1^	1.00 s*	1.00 f*	Self-selected	Multiple conditions
22	1.25 m·s^−1^	1.00 s*	1.00 f*	Self-selected	Multiple conditions
23	1.25 m·s^−1^	0.91 s*	1.10 f*	Self-selected	Fixed speed
24	1.25 m·s^−1^	0.83 s*	1.20 f*	Self-selected	Fixed speed
25	1.25 m·s^−1^	0.77 s*	1.30 f*	Self-selected	Fixed speed
26	1.25 m·s^−1^	1.00 s*	1.00 f*	0.0 m	Variable step width
27	1.25 m·s^−1^	1.00 s*	1.00 f*	0.1 m	Variable step width
28	1.25 m·s^−1^	1.00 s*	1.00 f*	0.2 m	Variable step width
29	1.25 m·s^−1^	1.00 s*	1.00 f*	0.3 m	Variable step width
30	1.25 m·s^−1^	1.00 s*	1.00 f*	0.4 m	Variable step width
31	1.40 m·s^−1^	1.00 s*	1.12 f*	Self-selected	Variable speed
32	1.40 m·s^−1^	Self-selected	Self-selected	Self-selected	Preferred walking
33	1.40 m·s^−1^	1.12 s*	1.00 f*	Self-selected	Variable step length

Most trials belong to one of five experimental conditions, and a few trials belong to multiple conditions. The preferred walking condition featured unconstraint walking, while other conditions had constraints on walking speed, step length, step frequency, or step width. The variable speed and variable step length conditions featured walking at various speeds with either step length or step frequency constrained to values preferred when walking at 1.25 m·s^−1^ (i.e., s* and f*, see Table 1). The fixed speed condition featured inversely varying combinations of step length and step frequency, while the variable step width condition featured various step widths at fixed speed, step length and step frequency. Trials 20–22 belong to all conditions, except for the variable step width condition.

### Experimental procedures

Participants were familiarized with a subset of trials during a 6-minute practice session before participating in the actual experiment. After performing a static standing trial for reference, the participant was asked to walk in a manner they preferred while the treadmill belt speed was set to 1.25 m·s^−1^. This first trial was used to determine the participant’s preferred step length s* and step frequency f* (see Table 1). Next, the participant practiced with a subset of trials, each lasting 30 s and together spanning the full experimental range (see Table 2). The order of practice trials was randomized for each participant individually. Next, the participant performed a series of 33 experimental testing trials, each for 60 s. Like with the practice trials, the participant was first asked to walk in a manner they preferred while the treadmill belt speed was 1.25 m·s^−1^. This first trial was used to reassess the participant’s preferred step length s* and step frequency f* (see Table 1). The preferred walking trial was repeated twice: once in the middle of the testing session, and once at the end. The order of the other 30 testing trials was randomized for each participant individually.

### Instrumentation and data collection

**Fig. 1:**
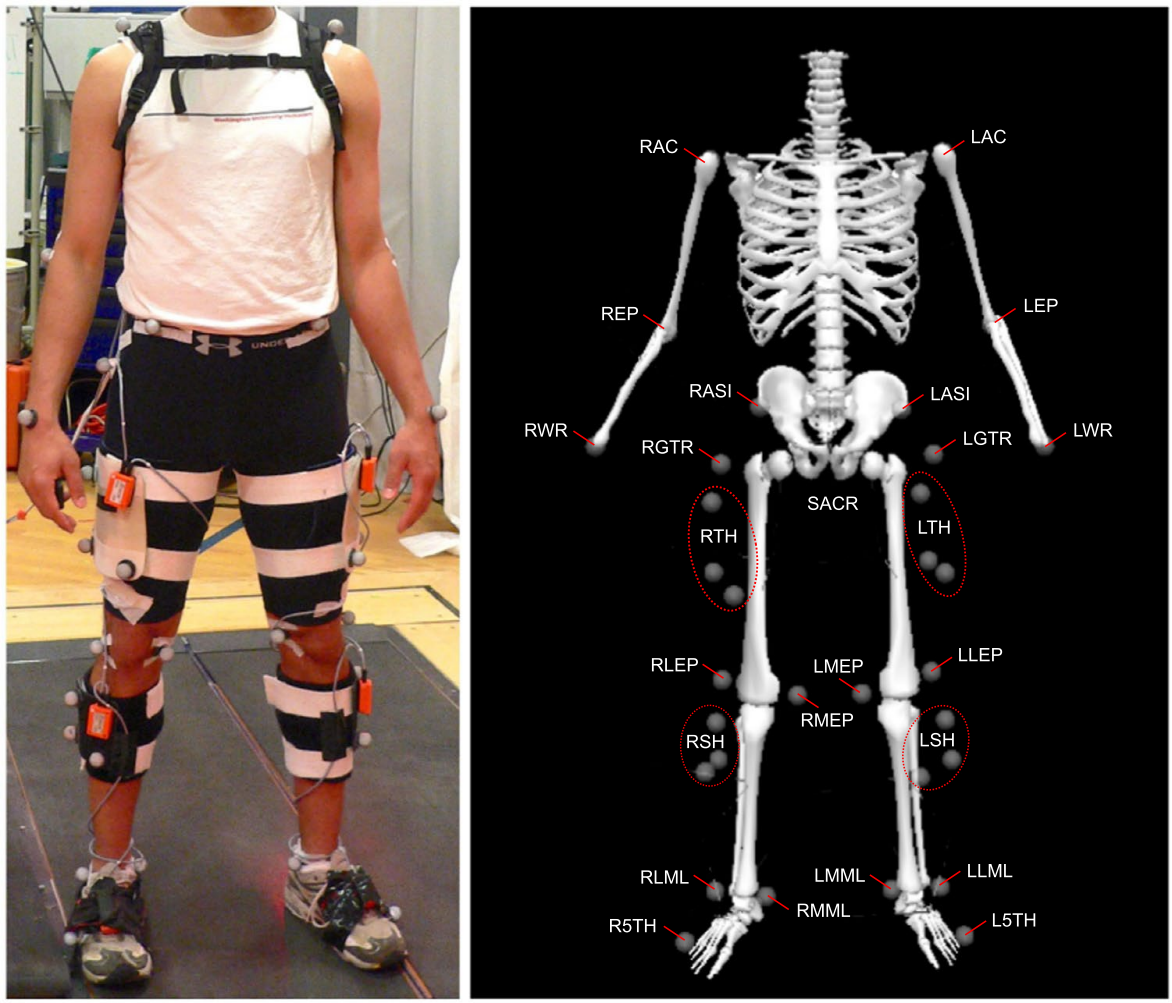
Marker locations on a human participant and in the inverse dynamic model. (**A**) Participant with motion capture markers attached to the limbs and anatomical locations. (**B**) Inverse dynamics model with estimated marker locations and modelled segment positions, visualized with a skeleton stick figure. Note that trunk and arm segments are shown here for illustrative purposes but were not used in inverse dynamics analysis due to the incomplete upper body marker set.

Kinematic and kinetic data were collected with standard gait laboratory procedures. Ground reaction forces were measured at 1200 Hz with two force platforms (Bertec, Columbus, OH, USA) located underneath a custom split-belt treadmill. Motion capture data was collected at 120 Hz using a standard 3D system (Motion Analysis Corporation, Santa Rosa, CA, USA), synchronously with the force platform recordings. Single markers were located at the head of the 5th metatarsus, calcaneus, malleoli, knee epicondyles, greater trochanter, anterior superior iliac spin, sacrum, acromion, elbow epicondyle, and wrist. Cluster markers were attached to the shanks and thighs. Virtual markers at Helen Hayes (Davis) points^10^ were estimated from the pelvis markers (see Fig. 1 and Table 3). Raw forces and motions were stored in C3D files.

**Table 3:** Marker locations.

Marker Name	Anatomical Location	Type	Inverse Dynamics	Segment or joint
LAC	Left acromion	Single	No	—
RAC	Right acromion	Single	No	—
LEP	Left elbow epicondyle	Single	No	—
REP	Right elbow epicondyle	Single	No	—
LWR	Left wrist	Single	No	—
RWR	Right wrist	Single	No	—
SACR	Sacrum	Single	Yes	Pelvis
LASI	Left anterior superior iliac spine	Single	Yes	Pelvis
RASI	Right anterior superior iliac spine	Single	Yes	Pelvis
HHL	Left Helen Hayes Davis point	Virtual	Yes	Pelvis
HHR	Right Helen Hayes Davis point	Virtual	Yes	Pelvis
LGTR	Left greater trochanter	Single	Yes	Left hip
RGTR	Right greater rochanter	Single	Yes	Right hip
LTH1	Left thigh marker 1	Cluster	Yes	Left thigh
LTH2	Left thigh marker 2	Cluster	Yes	Left thigh
LTH3	Left thigh marker 3	Cluster	Yes	Left thigh
RTH1	Right thigh marker 1	Cluster	Yes	Right thigh
RTH2	Right thigh marker 2	Cluster	Yes	Right thigh
RTH3	Right thigh marker 3	Cluster	Yes	Right thigh
LLEP	Left lateral knee epicondyle	Single	Yes	Left knee
LMEP	Left medial knee epicondyle	Single	Yes	Left knee
RLEP	Right lateral knee epicondyle	Single	Yes	Right knee
RMEP	Right medial knee epicondyle	Single	Yes	Right knee
LSH1	Left shank marker 1	Cluster	Yes	Left shank
LSH2	Left shank marker 2	Cluster	Yes	Left shank
LSH3	Left shank marker 3	Cluster	Yes	Left shank
RSH1	Right shank marker 1	Cluster	Yes	Right shank
RSH2	Right shank marker 2	Cluster	Yes	Right shank
RSH3	Right shank marker 3	Cluster	Yes	Right shank
LLML	Left lateral malleolus	Single	Yes	Left ankle
LMML	Left medial malleolus	Single	Yes	Left ankle
RLML	Right lateral malleolus	Single	Yes	Right ankle
RMML	Right medial malleolus	Single	Yes	Right ankle
LCAL	Left calcaneus	Single	Yes	Left foot
L5TH	Left 5th metatarsus	Single	Yes	Left foot
RCAL	Right calcaneus	Single	Yes	Right foot
R5TH	Right 5th metatarsus	Single	Yes	Right foot

Single markers were located at specific anatomical locations such as bony landmarks, cluster markers were located on the limbs, and virtual markers were inferred from single marker locations. Markers on the lower limbs were used in inverse dynamics analysis to estimate the locations of segments and joints, as well as the forces, torques and powers acting on them.

### Data analysis and export in Visual3D

**Fig. 2:**
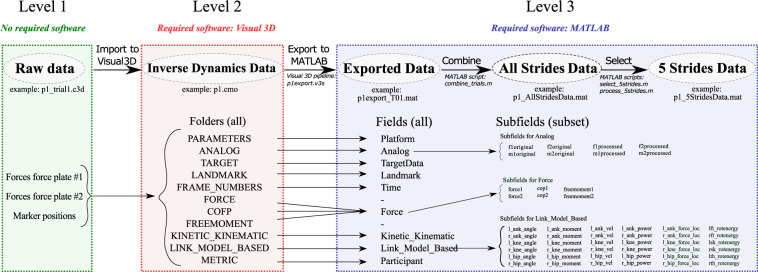
Workflow diagram of data processing. Raw data was processed with Visual 3D and MATLAB software to yield 5 strides data files. The required files for each step are shown in italics under the arrows. The folders and fields that contain the data in each step of the process are shown under the workflow.

Following storage in C3D format, collected forces and motions were filtered and processed with standard inverse dynamics software (Visual3D, C-Motion, Germantown, MD, USA). Visual3D software was first used to filter ground reaction forces and marker locations, employing a Butterworth low-pass filter with cut-off frequencies of 25 Hz and 6 Hz respectively, as in previous research^7^. Ankle, knee, and hip joints were defined based on locations of malleoli, epicondyles, and Helen Hayes (Davis) points respectively^10^. Joint angles were determined relative to a static standing trial. The offset in the ground reaction force was determined as the average over a manually selected time interval during which both feet were off the ground, and was subtracted from the ground reaction force signal. Inverse dynamics analysis was then performed on the processed ground reaction forces and marker locations of the lower limbs to obtain biomechanical variables, including 3D joint positions, forces, angles, torques, and powers. Filtered forces, motions and biomechanical variables were stored in CMO files. Finally, CMO data was exported to MAT files for further analysis with MATLAB software (MathWorks Inc., Natick, MA, USA), including selecting five strides of good quality (see Fig. 2).

## Data Records

The full dataset is available on figshare^11^. The data repository contains three folders, corresponding to three levels of data: (1) Raw data, including forces and motions in C3D format, (2) Inverse dynamics data, including computed biomechanical variables in CMO format, (3) Exported data, including biomechanical variables in MAT format (see Fig. 2). A subset of data level 3 (i.e., 5 strides data, see text below for description) has previously been made available as part of another publication^8^ and is also available on figshare^12^.

### Level 1: Raw data

Raw forces and marker locations are stored in public domain C3D format, which can be opened and processed using inverse dynamics software such as Visual3D. The C3D format is supported by all major 3D Motion Capture System manufacturers. Forces and marker locations may be used by themselves or combined in inverse dynamics analysis to compute biomechanical variables like joint torque and power. Each participant has one C3D file for each trial. For example, the C3D file for participant 1 trial 1 is called p1_trial1.c3d. All participants have 33 collected trials and therefore 33 C3D files, with one exception due to a missing C3D file (see Table 1). Table 2 indicates the experimental condition and gait parameters for each trial. For example, it shows that trial 1 belongs to the variable speed condition, with average speed $\bar{v}$ = 0.70 m·s^−1^ and step length *s* = s*.

### Level 2: Inverse dynamics data

Raw data were imported and processed using Visual3D software to yield inverse dynamics data, stored in (proprietary) CMO format. The CMO file is a combination of several C3D movement files, a C3D static calibration file, and a MDH biomechanical model description file. The model description file may be optionally changed by the user. Having multiple C3D movement files within one CMO file allows simultaneously applying a single biomechanical model to multiple trials. Each participant has one CMO file, containing all 33 trials. For example, the CMO file of participant 1 is called p1.cmo. Apart from data, the CMO file contains parameter values that can be set by the user. Data, parameter values, and model description together yield inverse dynamics variables, which can be expressed in local or global coordinate systems.

### Level 3: Exported data

Inverse dynamics variables were exported to MAT format, which can be opened using MATLAB software. We exported the variables through executing V3S pipeline scripts in Visual3D. A V3S pipeline script is an ASCII file that can be edited with any common word processing program or within the Visual 3D software. It instructs the Visual3D software to perform additional computation (e.g., filtering, coordinate transformation), and specifies which variables to export. Our V3S pipeline scripts call the Export_Data_To_Matfile Visual3D function to export data to MAT format. As an example, we have included a V3S pipeline script (p1export.v3s) in the software repository (see Code availability). After exporting each trial for each participant, we combined the trials into one file per participant. For example, the exported variables of participant 1 are stored in p1_AllStridesData.mat. Each file contains a 1 × 33 struct-array variable called “data” with fields: Platform, Analog, Target Data, Landmark, Time, Force, Kinetic_Kinematic, Link_Model_Based, and Participant. Each of these fields corresponds to one or more Visual3D folder(s) (see Fig. 2). In addition, we identified five strides of good quality and stored this in a separate data file. For example, the 5 strides data of participant 1 are stored in p1_5StridesData.mat. In most cases, there were many good strides within each trial, but some trials required selection to eliminate motion capture occlusions and to ensure that left-right steps landed on separate force plates.

## Technical Validation

We visualized, analysed, selected and compared data in MATLAB, and provide example scripts employed for these purposes (see Code availability). Overall, data was consistent within participants, across participants, and in comparison to existing datasets. On a few occasions, data appeared missing, inconsistent, or implausible, and we decided not to select five strides of good quality. Running the provided main.m script (see Code availability) results in recreating the 5 strides data files from the exported data files (see Fig. 2).

### Selecting five strides of good quality

Five strides may be selected with the MATLAB script ‘select_5strides.m’, which allows the user to select a five strides interval and visualize the five strides in comparison to the entire trial. The script outputs a MAT file called 5strides_heelstrikes.mat, containing the sample numbers that define the 5-strides interval. This MAT file is then used in another script called process_5strides.m to create the 5 strides data file.

### Analysis and visualization

**Fig. 3:**
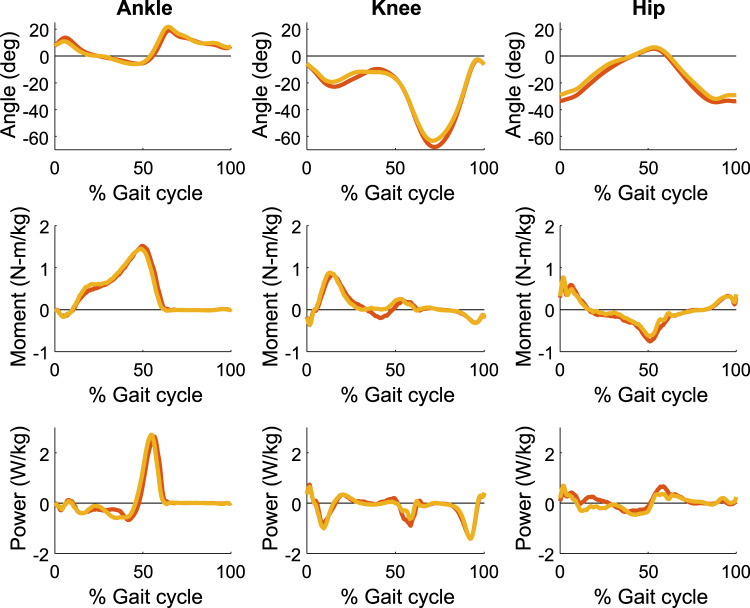
Typical example data of joint angle, moment, and power for ankle, knee, and hip. Lines indicate data from left leg (red) and right leg (yellow) of a representative participant (1), walking at nominal speed (1.25 m·s^−1^) with preferred step frequency and step length. For angle and moment, positive (negative) values indicate extension (flexion).

The MATLAB script ‘example_plotting.m’ may be used to investigate the 5 strides data for a specified biomechanical variable, participant and trial. It also creates a 3 × 3 plot of angle, moment and power for all three joints (i.e., ankle, knee, hip) (see Fig. 3). The MATLAB script ‘analyse_biomechanics_script.m’ calculates the mechanical work per stride at each joint and for the whole body. The user may clone the software repository and modify these scripts to accommodate their specific needs.

### Trials without five strides of good quality

There were a few occasions where we chose not to select five strides of good quality, including the variable step width conditions for all subjects, all trials for subject 10 and three individual cases.

#### Variable step width walking for all subjects

We did not select 5 strides data for variable step width walking (five trials for each subject). This condition included trials with zero or near-zero step width, where subjects did not generally step on distinct treadmill belts with each foot. Consequently, one of the two force plates measures the net effect of both limbs during double support, making it impossible to know the force acting on each limb. Individual limb inverse dynamics is therefore not appropriate for these trials and the computed biomechanical variables are unreliable. We advise to only use these trials when applying additional methods to estimate the force at each limb.

#### All trials for subject 10

We also did not select 5 strides data for subject 10, as comparison of biomechanical variables between subjects (within trials) consistently revealed this subject to be an outlier. Further inspection indicated that the modelled pelvis was disproportionally small, potentially due to incorrect marker placement. This may have resulted in an inaccurate hip joint centre location, resulting in physiologically implausible hip torques and powers. We advise to determine the hip joint centre location using methods that are independent of pelvis marker locations, for example using helical axis methods based on thigh marker locations.

#### Three individual cases

We did not select 5 strides data for three additional trials (out of the remaining 249). These were trials that showed large differences with the subject-mean, and with similar trials within the same subject. We discovered issues with these trials upon further inspection, and decided to exclude them from the 5 strides dataset. These trials belonged to one of two issue-categories (1) Synchronization error and (2) Incorrect stepping on force plates. These categories are discussed in more detail below.

1. *Synchronization error (two trials, different subjects)*
  Two trials (from different subjects) showed physiologically implausible joint powers that differed considerably from similar trials within the same subject, as well as from identical trials in other subjects. Upon closer inspection, these trials (subject 3, trial 4 and subject 9, trial 14) seemed to have a delay between recorded motions and forces. This was apparent from the discrepancy between the centre of pressure time-series (from recorded forces) and ankle- and toe marker location time-series (from recorded motions), which are closely related in other trials. We therefore decided not to select five strides for these trials. We advise to only use these trials after correctly synchronizing both signals, which should be done at the level of the individual C3D files.
2. *Incorrect stepping on force plates (one trial)*
  Like in the variable step width trials, one subject also consistently stepped on one of the two force platforms in one of their other trials (subject 4 trial 1). We therefore decided not to select five steps for this trial. Like with the variable step width trials, we advise to only use this trial when applying additional methods to estimate the force at each limb.

### Missing data

There were three trials (out of 330) with fully missing data and one subject with partially missing data, due to saving errors during data collection. In one of these cases (subject 7, trial 24), the C3D file was missing altogether. In another case (subject 6, trial 31), there was a C3D file, but it did not include marker locations (only forces). In the third case (subject 6, trial 21), data recording was stopped prematurely, and less than five strides were recorded. Lastly, the trials from subject 9 do not include the upper body marker positions of the acromion, elbow epicondyle, and wrist.

### Comparison to another dataset

**Fig. 4:**
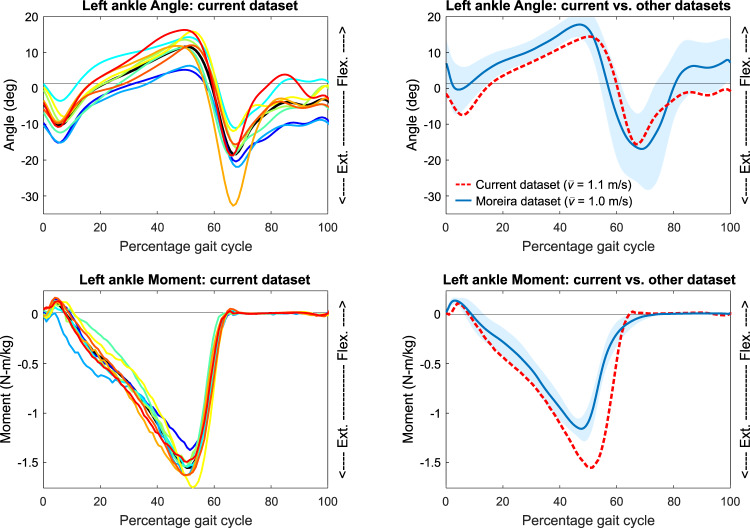
Representative data from present dataset compared to another dataset. Left column: Five strides of ankle data, during which participants walked at 1.1 m·s^−1^ with their preferred step length (preferred walking condition). Different colours represent different participants; the mean across participants is indicated by a black line (largely covered by the other lines). The ankle data is fairly consistent across participants. Right column: Current data (red dashed line) vs. data from another published repository^13^ (blue solid line). Present data agrees qualitatively with the data from a walking trial of comparable speed (1.0 m·s^−1^) obtained from the other dataset (mean indicated by solid line, s.d. indicated by shaded area).

Most trials showed relatively good consistency between participants, which may be verified by running the example_plotting.m MATLAB script for various biomechanical variables. For example, the participant-mean ankle angle and ankle moment were a reasonable representation of the individual participant data for preferred walking at 1.1 m·s^−1^ (see left column Fig. 4). In addition, these data show qualitative agreement with previously reported data for similar conditions. For example, the participant-mean ankle angle and ankle moment for preferred walking at 1.1 m·s^−1^ was comparable to those for preferred walking at 1.0 m·s^−1^ as reported in another (open-access) data descriptor paper^13^ (see right column Fig. 4). The consistency across both participants and datasets supports the validity of the current dataset.

### Limitations

The repeated-measures design of the experiment allows investigating effects of walking speed, step frequency, step length and step width on biomechanical variables such as mechanical work, force and joint moments. For example, previous studies have used this dataset to investigate how biomechanical variables like center of mass velocity^6^ and soft tissue work^7,8^ vary with gait parameters. While sufficient for investigating these particular effects, the sample size (N = 10) here may not be appropriate for some other applications. Further, it must be noted that because this dataset uses controlled, treadmill walking, its application should be limited to research questions regarding these applications. Considering biomechanical differences between treadmill and overground walking^14^ as well as between steady and non-steady walking^15^, the dataset does not address changes in self-selected, spontaneous walking speeds during overground walking^16^.

## Data Availability

Software is available on GitHub: github.com/timvanderzee/human-walking-biomechanics.
